# CRISPR/Cas9 and TALE: beyond cut and paste

**DOI:** 10.1007/s13238-015-0137-5

**Published:** 2015-02-27

**Authors:** Liping Deng, Ruotong Ren, Jun Wu, Keiichiro Suzuki, Juan Carlos Izpisua Belmote, Guang-Hui Liu

**Affiliations:** 1 National Laboratory of Biomacromolecules, Institute of Biophysics, Chinese Academy of Sciences, Beijing, 100101 China; 2 Gene Expression Laboratory, Salk Institute for Biological Studies, 10010 North Torrey Pines Road, La Jolla, CA 92037 USA; 3 Beijing Institute for Brain Disorders, Beijing, 100069 China; 4 Center for Molecular and Translational Medicine, CMTM, Beijing, 100101 China

**Keywords:** TALE, CRISPR/Cas9, genome editing, gene expression, transcription regulation

## Abstract

Nuclease-based genome editing has proven to be a powerful and promising tool for disease modeling and gene therapy. Recent advances in CRISPR/Cas and TALE indicate that they could also be used as a targeted regulator of gene expression, as well as being utilized for illuminating specific chromosomal structures or genomic regions.

Nuclease-based genome editing tools, including zinc finger nuclease (ZFN), transcriptional activator-like effector nucleases (TALENs), and the recently developed CRISPR (clustered regularly interspaced short palindromic repeat)–Cas system, have been progressing at an unprecedented pace for gene therapy and disease modeling (Gaj et al. 2013; Pan et al. 2011; Liu et al. 2014; Li et al. 2014). Recent advances have carved out new paths leading to novel applications of these genome engineering tools including visualization of specific loci of the genome and targeted regulation of gene expression.

In addition to their genome-editing capabilities, TALE and Cas9 have also been harnessed for targeted regulation of gene expression. Several studies exploited TALE and Cas9’s DNA binding abilities and converted them into synthetic transcriptional factors or epigenetic modifiers to modulate gene expression. Synthetic transcription factors created by fusing TALE or catalytically dead Cas9 (dCas9) to effector domains were successfully used to gain transcriptional control of gene expression. Binding of dCas9 to DNA alone could repress transcription (CRISPRi), possibly through stalling transcription elongation. Fusing dCas9 to protein domains that can recruit repressive chromatin-modifying complexes, e.g. the KRAB domain of Kox12, can further enhance CRISPRi. To activate genes, in one study Therizols et al. (2014) fused TALE to VP64, a tetramer of the VP16 acidic transcriptional activator and used the fusion protein to ectopically activate genes normally silenced, leading to novel insights of nuclear reorganization in embryonic stem cells (ESCs). Similarly, Gilbert et al. fused dCas9 to the activation domains of either VP64 or p65 to activate targeted genes (Gilbert et al. 2013). Synthetic epigenetic modifiers were created in Maeder et al.’s study (2013) by fusing a hydroxylase catalytic domain of TET1 to TALE, leading to targeted demethylation of specific promoter CpGs. Removal of the methylation from key promoter CpGs can result in enhanced transcription of endogenous genes. Tethering Tet1 hydroxylase domain to a target promoter thus constitutes a proof-of-concept in epigenetic activation of specific gene transcription. To epigenetically repress transcription of target genes, Mendenhall et al. (2013) fused TALE with the LSD1 histone demethylase which enabled targeted demethylation of enhancer-associated histone modifications thereby repressing the proximal genes. Furthermore, a recent study achieved spatiotemporal transcriptional regulation by combining TALE and a popular optogenetic approach and created a LITE (light-inducible transcriptional effectors) system (Konermann et al. 2013). The LITE system includes two parts: a customizable TALE DNA-binding domain, fused with light-sensitive cryptochrome 2 (CRY2), and transcriptional regulator-fused CIB1 (an interacting partner of CRY2). The LITE system allows for precise spatiotemporal control of genetic and epigenetic factors contributing to a variety of biological processes *in vivo*. Very recently, two studies further expanded the application of CRISPR/Cas9 to genome-wide interrogation of gene function through systematic transcriptional perturbation. One study by Zalatan et al. (2014) incorporated modular RNA domains into the sgRNA design and repurposed sgRNA as scaffolding molecules that encode both target and function in a single scaffold RNA (scRNA). scRNA enables simultaneous multidirectional regulation of multiple target genes. In the second study, Konermann et al. (2014) showed that following structure-guided design, a novel CRISPR–Cas9 complex could drive transcriptional activation at endogenous gene loci efficiently. Moreover, genome-wide transcriptional activation was realized with a library composed of 70,290 engineering guides targeting all coding isoforms of human RefSeq, with which they screened genes exhibiting resistance to a BRAF inhibitor upon activation.

Interestingly, TALE and Cas9 have also been brought to service to visualize specific genomic loci in live cells, which provide a novel way to uncover the functional relevance between chromatin spatial organization and genome function. Traditionally fluorescent *in situ* hybridization (FISH) is the method of choice to label DNA. However, FISH requires sample fixation and is incompatible for monitoring live cellular processes. Three recent studies turned TALE or Cas9 into powerful live cell imaging tools. In Miyanari et al.’s (2013), fluorescent TALE was designed to visualize major satellites in cultured mouse cells, including centromeric and telomeric elements in the genome. Moreover, the TALE-based approach exhibited high specificity allowing for distinguishing single-nucleotide polymorphisms (SNPs). Ma et al. (2013) successfully labeled telomeres in human cells by fusing fluorescent protein (FP) to a TALE targeting a telomeric sequence. They also designed unique centromeric sequences specifically associated with certain chromosomes to visualize individual chromosomes in human cells. Meanwhile, since signals generated by FP-TALE positively correlated with telomere length, they could measure telomere length in human cells. Results from the above methods are consistent with those obtained by DNA-FISH, suggesting their potential in labeling specific genomic sequences with high accuracy. Therefore, TALE-based strategies hold immense promise to gain insight into the chromatin dynamics associated with different cellular physiologies by visualizing genomic DNA repetitive sequences. Importantly, an optimized CRISPR-Cas system with structure-guided sgRNA was recently employed for efficiently labeling arbitrary genomic sequences in live mammalian cells (Chen et al. 2013). It proved to be a robust method for imaging of both repetitive elements and coding genes. Although genome-scale imaging has not been implemented with a library of sgRNA, visualization of specific genomic loci paves the way for further study of dynamic organization of the human genome.

The advances in targeted genome engineering technologies via CRISPR/Cas9 and TALE may lead to a revolution in cell biology research. Several considerations nevertheless should be taken into account for future applications. (1) Whether potential off-target effects can contribute to certain bias or misinterpretation in obtained results. While the current whole-genome sequencing has indicated that gene editing mediated by well-designed sgRNA and TALEN leads to minimal mutational load at global level (Hsu et al. 2013; Smith et al. 2014; Suzuki et al. 2014; Veres et al. 2014), precautions need to be taken to minimize off-targets caused by poor sgRNA and TALE design. (2) Choice of TALE vs Cas9 in specific application? From the aspect of construct size, TALE is relatively smaller than Cas9 and is easier to be delivered into cells; engineered TALE may be advantageous in site-specific transcriptional manipulation by serving as a direct transcriptional regulator or, alternatively, establishing a bridge between protein and DNA or between two genomic DNA loci without involving exogenous gRNA molecules. By contrast, while Cas9 with larger size is difficult to get into cells, it holds the potential for multiplex targeting. (3) It still remains unclear whether binding of TALE or Cas9–gRNA complex to the target sites would perturb the function of the native DNA-binding proteins and cause unwanted perturbation of cellular function. (4) Whether intra-nuclear localization, three-dimensional organization, or epigenetic modifications of target DNA element potentially affect the binding of engineered TALE and Cas9–gRNA warrants further investigations. Also how these factors contribute to unbiased interpretation of the observed results needs to be considered.

Collectively, despite some current concerns for using engineered Cas9 and TALE for exploring uncharted cellular events, esp. in a physiologically relevant context, these tools undoubtedly open a new avenue to uncover novel events underlying gene expression regulation and dynamic nuclear organization of chromatin, and may greatly facilitate translation of basic studies into clinical therapies.
